# A Novel Transcriptome Integrated Network Approach Identifies the Key Driver lncRNA Involved in Cell Cycle With Chromium (VI)-Treated BEAS-2B Cells

**DOI:** 10.3389/fgene.2020.597803

**Published:** 2021-01-13

**Authors:** Pai Zheng, Yulin Kang, Shuo Han, Huimin Feng, Feizai Ha, Changmao Long, Di Zhou, Guiping Hu, Zhangjian Chen, Zengmiao Wang, Tiancheng Wang, Guang Jia

**Affiliations:** ^1^Department of Occupational and Environmental Health Science, School of Public Health, Peking University, Beijing, China; ^2^Institute of Environmental Information, Chinese Research Academy of Environmental Sciences, Beijing, China; ^3^School of Medical Science and Engineering, Beihang University, Beijing, China; ^4^State Key Laboratory of Remote Sensing Science, College of Global Change and Earth System Science, Beijing Normal University, Beijing, China; ^5^Department of Chemistry and Biochemistry, University of California, San Diego, La Jolla, CA, United States

**Keywords:** hexavalent chromium, transcriptome, cell cycle, lncRNA, regulation network

## Abstract

Hexavalent chromium [Cr(VI)] is a well-known occupational carcinogen, but the mechanisms contributing to DNA damage and cell cycle alternation have not been fully characterized. To study the dose-response effects of Cr(VI) on transcription, we exposed BEAS-2B cells to Cr(VI) at concentrations of 0.2, 0.6, and 1.8 μmol/L for 24 h. Here, we identified 1,484 differentially expressed genes (DEGs) in our transcript profiling data, with the majority of differentially expressed transcripts being downregulated. Our results also showed that these DEGs were enriched in pathways associated with the cell cycle, including DNA replication, chromatin assembly, and DNA repair. Using the differential expressed genes related to cell cycle, a weighted gene co-expression network was constructed and a key mRNA-lncRNA regulation module was identified under a scale-free network with topological properties. Additionally, key driver analysis (KDA) was applied to the mRNA-lncRNA regulation module to identify the driver genes. The KDA revealed that ARD3 (FDR = 1.46 × 10^–22^), SND1 (FDR = 5.24 × 10^–8^), and lnc-DHX32-2:1 (FDR = 1.43 × 10^–17^) were particularly highlighted in the category of G2/M, G1/S, and M phases. Moreover, several genes we identified exhibited great connectivity in our causal gene network with every key driver gene, including CDK14, POLA1, lnc-NCS1-2:1, and lnc-FOXK1-4:1 (all FDR < 0.05 in those phases). Together, these results obtained using mathematical approaches and bioinformatics algorithmics might provide potential new mechanisms involved in the cytotoxicity induced by Cr.

## Introduction

Inhalation exposure to hexavalent chromium [Cr(VI)] has been recognized as a significant occupational carcinogen according to the final document from the National Institute for Occupational Safety and Health (NIOSH) (NOISH, 2008). Several researchers have explored the underlying molecular mechanisms induced by Cr(VI) related to cellular transformation (Karaulov et al., 2019) and tumorigenesis (Holmes et al., 2008; Wang et al., 2019), whereas the transcriptomic responses remain elusive. Of all the possible mechanisms of carcinogenesis associated with exposure to Cr(VI), it is hard to ignore the effects of epigenetic modifications and cytogenetic damage (Rager et al., 2019), mainly the regulatory signaling pathways related to the two processes described above.

To better understand the history of transcriptome studies in the field, a brief bibliometric study was conducted to identify trends based on the frequently occurring keywords in published papers from different years. This study clearly demonstrated the changes in the main topics in this area during the last half-century. Since the first report of cancer among a population of Cr-exposed workers, Cr(VI) compounds have remained on the list of potential threats to human health. Before 1995, several studies laid a profound foundation in the toxicity field, especially regarding DNA expression under the condition of exposure to multiple metals (i.e., with nickel) (Alcedo et al., 1994) and the proposed “uptake-reduction” model, which suggests the hypothesis that molecular events of genes may induce gene expression changes in carcinogenicity (Wetterhahn and Hamilton, 1989; Dubrovskaya and Wetterhahn, 1998) and DNA damage (Standeven and Wetterhahn, 1989). From 1995 to 2000, researchers mainly focused on the toxicology of lung cells, including type II pneumocytes (Shumilla and Barchowsky, 1999) and fibroblasts (Carlisle et al., 2000), and the mechanisms of apoptosis induced by genetically programmed cell death or the effect of transcriptional inhibition (Singh et al., 1998; Röpke et al., 2000). From 2000 to 2005, Cr-induced apoptosis became the most debatable topic. Researchers studied the possible sensors or mediators involved in apoptosis, particularly the effect of ATM protein and p38 MAP kinase (Ha et al., 2003; Wakeman et al., 2005). In addition, the role of free radicals following Cr(VI)-induced DNA damage and carcinogenesis was speculated (Liu et al., 2001; Zhang et al., 2001). From 2006 to 2010, the potential mechanisms of Cr(VI) carcinogenesis in lung cells were extensively published, and the main hypothesis was related to genomic instability (Holmes et al., 2008), including microsatellite instability (Hirose et al., 2002; Takahashi et al., 2005), numerical chromosome instability (Xie et al., 2005), and consequences of the imbalance between cellular damage and repair systems (Yao et al., 2008). Then, by the year 2010, the Environmental Working Group had detected Cr-polluted drinking water in 42 states that affected 74 million Americans (Sutton, 2010), resulting in calling for a legal limit for Cr(VI) and studies investigating the environmental carcinogenicity targeted toward the digestive system (Stern, 2010; Kopec et al., 2012; Thompson et al., 2014). Additionally, the successful discovery of microRNAs (miRNA) (Hobert, 2008) provided a new perspective for gene regulation research. As a result, miRNA studies related to Cr were introduced into the field of environmental toxicology, and they aimed to elucidate the mechanisms of lung cancer induced by Cr (He et al., 2013; Li et al., 2014). Over the last 4 years, miRNA-related research has been a topic of high interest and has recently received increasing attention, especially topics related to DNA repair (Li et al., 2016) or glycolipid metabolism (Zhang et al., 2018).

Although all of these changes have occurred, studies investigating the genotoxic impact of Cr are still emerging, and Cr(VI) carcinogenicity is widely debated (Zhitkovich, 2011). In particular, the roles of non-coding RNAs in transcriptional responses during the exposure of physiological and toxicological levels are not well understood. Therefore, we conducted a toxicogenomics study using a data-driven analysis approach that aimed to outline integrated networks and to identify candidate key driver genes involved in the underlying mechanisms of cell cycle alterations after Cr exposure. Furthermore, considering the widespread involvement of long non-coding RNAs (lncRNAs) in multiple cellular functions (Mercer et al., 2009; Yao et al., 2019), including the cell cycle (Kitagawa et al., 2013) and other Cr-induced processes (Hu et al., 2019), we hypothesized that lncRNAs and coding genes might be key mediators of the responses to DNA damage by regulating the cell cycle in Cr-induced genotoxicity.

For the purpose of building a network to reveal the correlations between these RNAs, we integrated the results of several bioinformatic analysis approaches based on the expression data of RNAs. These included weighted correlation network analysis (WGCNA) to create modules according to highly correlated gene expression patterns (Langfelder and Horvath, 2008). Moreover, to pinpoint the key driver gene in these processes, we conducted weight key driver analysis (Shu et al., 2016) to extensively search for potential key elements in the regulation network (Sun et al., 2015; Bailey et al., 2018) and to detect the possible trigger genes in different phases of the cell cycle. Thus, our approach aimed to provide a candidate gene list for further research on explaining the underlying molecular mechanisms that regulate the cell cycle following exposure to relatively low concentrations of Cr(VI), and to identify the non-coding RNAs that might be novel candidate molecular targets for exposure biomarker studies.

## Materials and Methods

### Cell Culture and RNA Extraction

The human bronchial epithelial cell line (BEAS-2B) was purchased from American Type Culture Collection (ATCC, United States), maintaining at 37°C and 5% CO_2_ in a humidified incubator. Cells were cultured in Bronchial Epithelial Cell Growth Medium (BEGM^TM^, BulletKit^TM^Lonza, Switzerland) supplemented with the necessary components and growth factors. The BEGM media was replaced every second day, and cells were passaged when they reached 70–80% confluency by incubation with 0.25% trypsin. In the presence of a diluted potassium dichromate stock solution (K2Cr2O7, Sigma, United States), BEAS-2B cells were seeded in six-well plates (105 cells/well) and exposed to low (0.2 μmol/L), medium (0.6 μmol/L), and high concentrations (1.8 μmol/L) of Cr(VI) for 24 h. A control group was established under the same conditions as the exposure groups. Each sample containing approximately 1 × 10^7^ cells was disrupted in buffer RLT (Qiagen, United States) for RNA isolation. Total RNA was isolated using the miRNeasy Mini Kit (Cat#217004, QIAGEN, GmBH, Germany) according to the manufacturer’s recommended guidelines, and the RIN number was determined to analyze RNA integrity using an Agilent Bioanalyzer 2,100 spectrophotometer (Agilent Technologies, Santa Clara, CA, United States). To assess the purity of RNA, a NanoDrop 2000c (Thermo Fisher Scientific, United States) and UVP Imaging System were used to measure the 260/280 ratios. Samples with a RIN number > 7 and 260/280 ratio in the range of ∼2.0 were considered qualified samples.

### RNA Isolation and RNA Microarray

Total RNA was amplified and labeled using a Low Input Quick Amp Labeling Kit, One-Color (Cat.# 5190-2305, Agilent Technologies). Each slide was hybridized with 1.65 μg of Cy3-labeled cRNA with a Gene Expression Hybridization Kit (Cat.# 5188-5242, Agilent Technologies), maintaining in a Hybridization Oven (Cat.# G2545A, Agilent technologies) for 17 h hybridization. Then, slides were washed with the Gene Expression Wash Buffer Kit (Cat.# 5188-5327, Agilent Technologies) and scanned by an Agilent Microarray Scanner (Cat#G2565CA, Agilent Technologies). Finally, data were extracted with Feature Extraction software 10.7 (Agilent Technologies). Raw data were normalized with the quantile algorithm and limma packages in R.

### Differential Expression Analysis

The normalization of gene expression and differentially expressed (DE) gene analysis were performed by functions in R package limma (Ritchie et al., 2015). Briefly, the function lmFit was used to fit a linear model to estimate the variability in the data. Using the function eBayes in limma, the significance of differences in the variance of gene expression across biological replicates for each gene were calculated using empirical Bayes moderated t-statistics tests. For the multiple hypothesis testing correction, the false discovery rate (FDR) was applied. The fold change in logarithm form between the samples from treated group and the samples from control group was also calculated for each gene (Smyth, 2005). Finally, genes were defined as DE if FDRs were below 0.05 and |log2FC| ≥ 1.

### Cell Cycle-Related Genes

After analyzing the pathways, significantly expressed pathways were identified with the cutoff of adjusted *P* < 0.05. From our results, we observed that significant pathways were related to biological processes involved in the regulation of the cell cycle, which had 11 related pathways. Furthermore, we extracted all genes with the GO annotations of the cell cycle (GO:0007049) from AmiGO 2 (Carbon et al., 2009) and withdrew the DE genes in our database. At last, a linear regression was performed with the expression of each gene. We used the 12 values in 4 dose groups (each dose had triplicated biological samples) as the dependent variable, and the dose 0–1.8 μmol/L corresponding to each sample as the independent variables. We took the linear regression slope to represent expression-dose-depend relationship for each gene and filtered the genes with a threshold of the median coefficient from the whole gene set. The g:Profiler (Reimand et al., 2016) (database built on 2020-03-07) was used to conduct GO enrichment analysis based on the DE gene list identified from the high dose group vs. with the control group comparison. We also visualized the g:GOSt (database built on 2020-03-07) enrichment results from different annotation resources, including GO, KEGG, REAC, and TF.

### Co-expression Network Detection Using WGCNA

WGCNA (Langfelder and Horvath, 2008) was used to identify the co-expression module with the selected cell cycle-related coding RNAs. WGCNA is a guilt-by-association approach for constructing networks and module detection. We computed a correlation raised to a power as a soft thresholding between every pair of RNAs to amplify the disparity and transformed the result into an adjacency matrix. Then, the blockwiseModules function was used to compute topological overlap matrix (TOM) dissimilarity (Yip and Horvath, 2007) between genes, and a hierarchical clustering gene dendrogram was constructed. According to the standard of dynamicTreeCut, modules whose eigengenes are highly correlated are merged with the threshold of 0.2. At last, we applied the module preservation analysis to test whether the features of each module were preserved in an alternative set of samples.

The preservation analysis is to estimate the differences between the observation in our gene expression and random situations by permutation (nPermutations = 10). Furthermore, Z-summary statistical analysis was performed as a general summary of all of the different statistics used, and scores > 10 were considered preserved (Langfelder et al., 2011).

### Key Driver Analysis

To conduct key driver analysis, we used the R package KDA (v1.14.0) (Shu et al., 2016). The purpose of KDA analysis was to analyze the detailed interactions between coding genes and lncRNAs to select genes that were over-represented in the regulation of the cell cycle. The package first required a sub-network file and gene list of interest as input files. Then, the enrichment in the k-step downstream neighborhood for the target gene list was assessed in the sub-network. In this study, we used a list of cell-cycle related genestarget list as the input file. We computed the co-expression pairs between targeted coding genes and all lncRNAs in our experimental study as the network file. We divided these coding genes into three group according to the key events in distinct phases, for example the checkpoint which regulated the transition of phases. Finally, we combined the lists with the name of cell cycle class as the input lists for KDA detection. The KDA results were visualized using Cytoscape (v3.7.2).

### Quantitative Real-Time Polymerase Chain Reaction (qRT-PCR)

For quantitative real-time PCR (RT-qPCR) analyses, total RNA was isolated from the control, middle, and high dose groups of the samples as described above, and those groups along with three independent replications of the biological experiments were analyzed. We selected and verified the key driver genes and genes in the network (Figure R1) for their biological potential in explaining the Cr(VI) cytotoxicity with the following criteria: (1) the genes participate in regulating at least two stages of cell cycle progress in the subnetwork; (2) the genes were reported to be involved in the biological functions, such as cell cycle arrest, carcinogenicity, and lung cancer, according to the literature. According to these criteria, we selected three groups of genes, in the (1) PARD3 group: ENST00000607815 and PARD3B, (2) SND1group: TEX14, (3) genes in both PARD3, and SND1 group:lnc-NCS1-2:1, FBXO6, CDK14, lnc-DHX32-2:1, lnc-FOXK1-4:1, POLA1, NR_002579, lnc-C3orf14-1:1 as the target genes for further validation.

Primer sequences were designed using Primer Express 3.0.1 designer software and then verified with NCBI Primer-BLAST software to confirm specific recognition of the target lncRNAs and mRNAs. qPCR was performed using a CFX96 Touch Real-Time PCR Detection System (Bio-Rad Laboratories, United States) to determine the expression levels of selected genes, and the results were presented as the mean value for the three wells. Data were calculated using the 2^–△△^*^*Ct*^* with glycerol-3-phosphate dehydrogenase (GAPDH) as the endogenous control.

## Results

### DE Genes Between Different Groups

**FIGURE 1:**
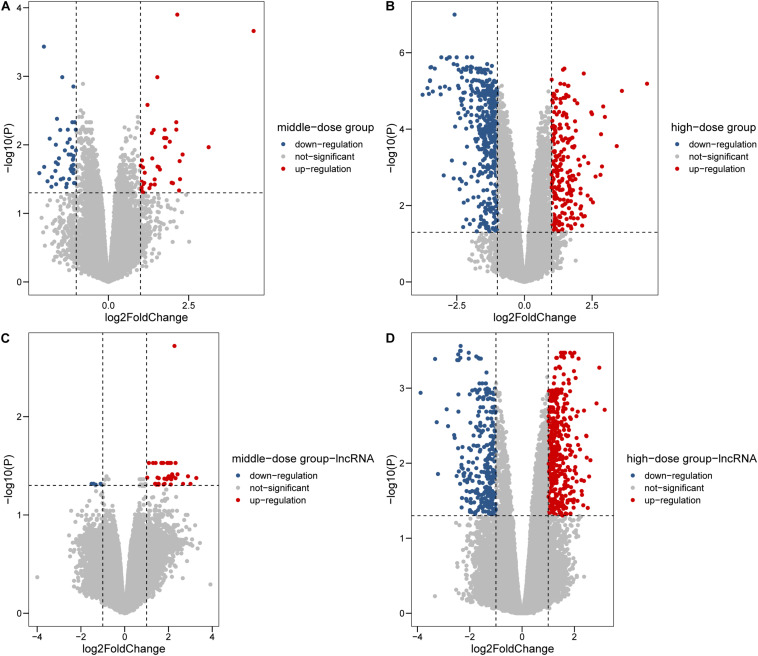
Differentially expressed genes in 0.6 and 1.8 μmol/L Cr(VI) groups. **(A,B)** Differentially expressed mRNAs in 0.6 and 1.8 μmol/L Cr(VI) groups. **(C,D)** Differentially expressed lncRNAs in 0.6 and 1.8 μmol/L Cr(VI) groups.

In total, we collected 12 samples, 3 from the low-dosage group, 3 from the medium-dosage group, 3 from the high-dosage group and 3 from the control group. Genome wide analysis of RNA expression by microarray was used to examine the transcription changes between the Cr(VI) treated group and the control group. To fully characterize the regulation pattern between mRNA and lncRNA, 18833 mRNA and 68104 lncRNA were considered in this study. Differential expression analysis was conducted for each Cr(VI) dosage vs. control comparison. The largest differences in gene expression were evident in the high dose group (1.8 μmol/L) with log2 fold-change [log2(FC)] values ranging from −3.76 to 4.52, including 646 upregulated and 771 downregulated genes (Figures 1B,D). A relatively lower number of differentially expressed genes was observed in the middle (129) (Figures 1A,C) and low dose (17) groups compared with normal cultured cells. It was evident that there were more upregulated and downregulated lncRNAs in the 1.8 μmol/L group compared with the other groups (Figure 1C), and DE lncRNA from high-dose accounts for 89.3% of all DE lncRNAs across three groups. From the histograms of fold change profile for mRNA and lncRNA in the two groups, we found that the high dose group had more differentially expressed transcripts with log2FC values above 0.5 and below −0.5 than the middle dose group (Supplementary Figure 1A), whereas the differences in lncRNAs were not as clear (Supplementary Figure 1B). Taken together, the high level of hexavalent chromium induces the dramatic changes in transcription profiling. To get a unified DE gene set, we merged the DE genes from three different groups and 1,484 mRNAs and lncRNAs were left (Supplementary Table 1), while the detail information was listed in Supplementary Tables 2, 3.

### Enrichment of Protein-Coding Genes

**FIGURE 2:**
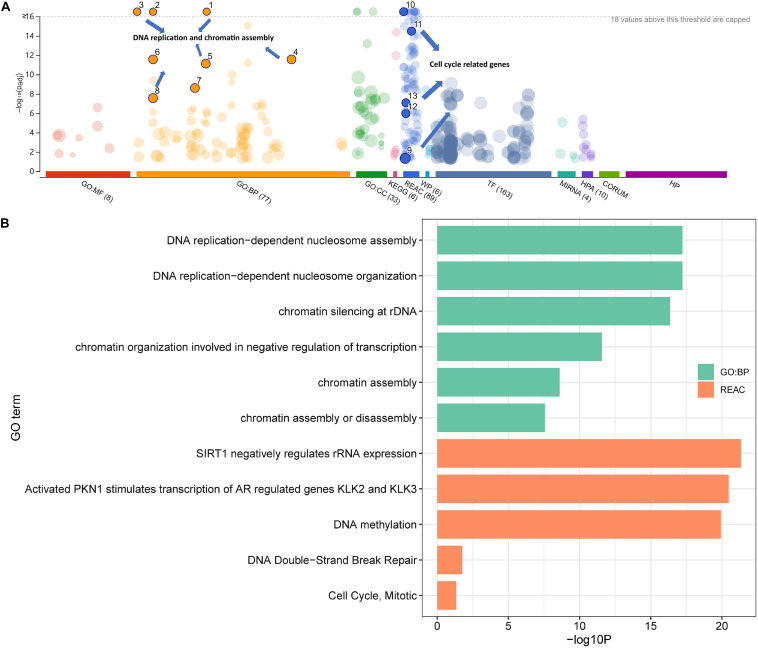
Enrichment analysis of differentially expressed genes in the high-dose group. The total transcript level correlates to **(A)** cell cycle processes and **(B)** detailed information for the chosen pathways.

To further explore the potential biological processes induced by Cr, enrichment analyses using DEGs databases were conducted using g:Profiler. As shown in Figure 2, DNA replication (FDR = 5.97 × 10^–18^) and chromatin organization (FDR = 2.70 × 10^–12^) from GO biological process database, cell cycle-related pathways from the Reactome Pathway Database (REAC) were identified with almost the highest statistically significant confidence level (all FDR < 0.05), suggesting that these processes might substantially change after exposure to the Cr(VI) (Figure 2B). Moreover, after analyzing the most altered pathways in replicating cells in detail, we identified the pathway involving SIRT1 (FDR = 4.56 × 10^–22^), a component of the Energy-dependent Nucleolar Silencing Complex (eNoSC), which may serve as an important integration point in cell cycle regulation (Bouras et al., 2005). Other pathways, including DNA double-strand break repair (FDR = 0.017) and mitotic cycle alternation (FDR = 0.046), also suggested a link between the modification of the cell cycle and exposure to Cr(VI). Therefore, we selected genes with strong enrichment with specific processes as the target biological reactions for analysis.

### Selection of Cell Cycle-Related Genes

To determine the gene list for further analysis of transcript profiles and co-expression with lncRNAs, we select cycle-related genes based on three criterion: (1) genes annotated with the function of cell cycle, (2) DE genes, (3) existing linear relationship. We first downloaded the coding gene data and their information regarding GO class and evidence code from AmiGO 2 with the annotation of the cell cycle (GO:0007049) as the background list. Subsequently, 64 gene transcripts were matched with our differentially expressed genes, and the list in AmiGO 2 of 918 genes was related to 1,952 items with target processes. These genes were then screened according to the criterion for dose-expression relationships. To accurately identify the genes related to the effect of Cr(VI) treatment on cell proliferation, we filtered genes with absolute regression coefficients > 0.4 (close to the value of Q1 and Q3 in the cell cycle). Finally, 35 genes were selected as targeted mRNAs for the construction of co-expression modules with lncRNAs.

**FIGURE 3:**
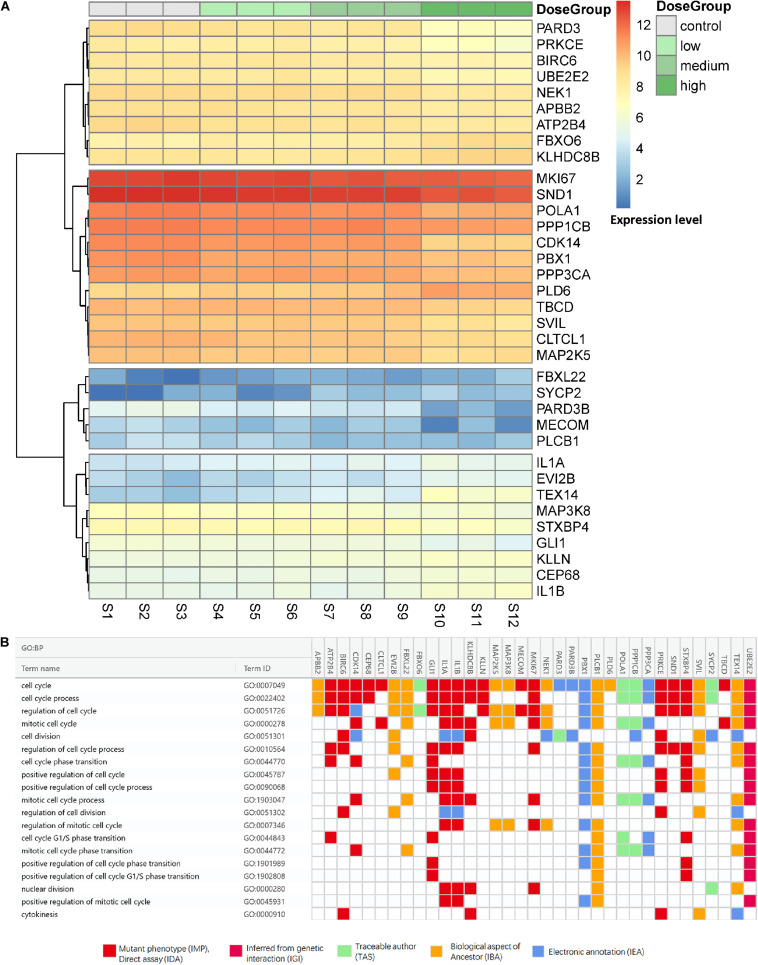
Expression levels and evidence codes of target cell cycle genes. **(A)** The expression level of the cell cycle-related genes in different groups and samples and **(B)** the gene ontology evidence codes of the selected genes in the cell cycle.

The expression levels in the four treatment groups from the mRNA profiles and evidence codes of these genes in the cell cycle are shown in Figure 3. As shown in Figure 3A, genes were clustered into four groups, even with similar pathways in the process of regulation of the cell cycle in all clusters, we still found the cluster-specific pathway, indicating that Cr(VI) may affect the regulatory functions in different aspects. In particular, cluster one was enriched in the function of cell division (*P* = 1.16 × 10^–6^), whereas cluster two focused on mitotic cell cycle phase transition (*P* = 6.11 × 10^–6^), especially the G2/M transition of the mitotic cell cycle (*P* = 2.30 × 10^–4^). The last cluster with 9 genes, including IL1A and IL1B, was associated with the regulation of cell cycle processes (*P* = 3.76 × 10^–5^). Additionally, as shown in Figure 3B, 17 of 35 gene annotations were derived from laboratory experiments, suggesting that the transcripts were closely related to the process of cell cycle regulation.

### Co-expression Module With Cell Cycle-Associated Protein-Coding Genes and lncRNAs

**FIGURE 4:**
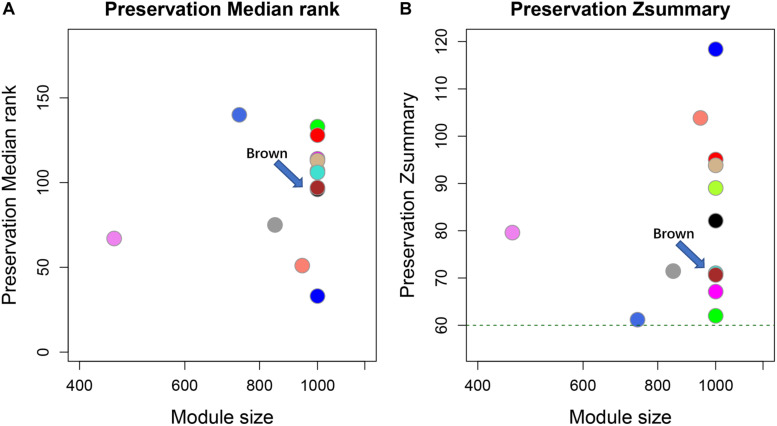
Module preservation test for co-expression cell cycle mRNA data sets. Circles in both figure represent different co-expression modules. The horizontal and vertical axes represent gene number and Z_*summary*_ values for each module with random selection, respectively. The green dotted line indicates Z_*summary*_ = 60 (Z_*summary*_ > 10 indicates high preservation). **(A)** Preservation median rank of these modules. **(B)** Preservation Z_*summary*_. Brown modules were indicated by arrows.

To investigate lncRNAs that were highly connected to a given set of interesting protein-coding genes, WGCNA was employed. WGCNA is R software package to perform network construction, module detection, and calculation of topological properties based on the guilt-by-association strategy. The WGCNA network was generated using the selected 35 mRNA and all lncRNA profiles from the different dose groups. We selected 16 as the soft threshold based on the results of scale-free topology and mean connectivity for the construction of the network. All genes were clustered into a unique module and the cluster dendrogram shown in Supplementary Figures 2, 3. Altogether, we identified six modules that contained target cell cycle protein-coding genes from all 185 co-expression modules. Modules were color-coded as brown, plum1, black, gray60, magenta, and tan4 with 25, 5, 2, 1, 1, and 1 mRNA, respectively. A preservation test was applied to confirm the reliability and sensitivity of the results using a WGCNA function (modulePreservation). Briefly, to evaluate whether a module was conserved or not, the Zsummary (Z-score) was calculated. All six modules were highly preserved with Z-scores > 30, whereas the brown (Z_summary_ = 68) was chosen for obtaining the highest number of transcripts (Figure 4). Therefore, modules colored brown containing 25 mRNAs and 3,565 lncRNAs were regarded as highly representative modules according to the expression patterns of these genes, and served as a cell cycle-regulated network for further analysis.

### Key Driver Analysis

Previous analysis recommended that genes Within the brown module Were the key cell cycle regulatory gene set. We performed key driver analysis (KDA) for these target gene co-expression modules using our previously published method (Shu et al., 2016). Key driver genes or key regulatory components were defined as the subnetworks with respect to various biological contexts, when compare to the other random genes in the network. The KDA requires a gene list (generally a biological-associated gene list) and a gene network as input files to identify the key genes. Under different cell cycle phases, we first divided 25 mRNA in the brown module Into three categories (G1/S, G2/S, and M) according to their GO subcategory annotation (with the highest level of evidence code in each gene), which were connected to the biological regulatory events in cell cycle processes. Then, Pearson’s correlation coefficient was calculated to each selected protein-coding gene and all differentially expressed lncRNAs. Pairs with absolute values of Pearson’s correlation coefficients ≥ 0.90 were selected as the network file.

**FIGURE 5:**
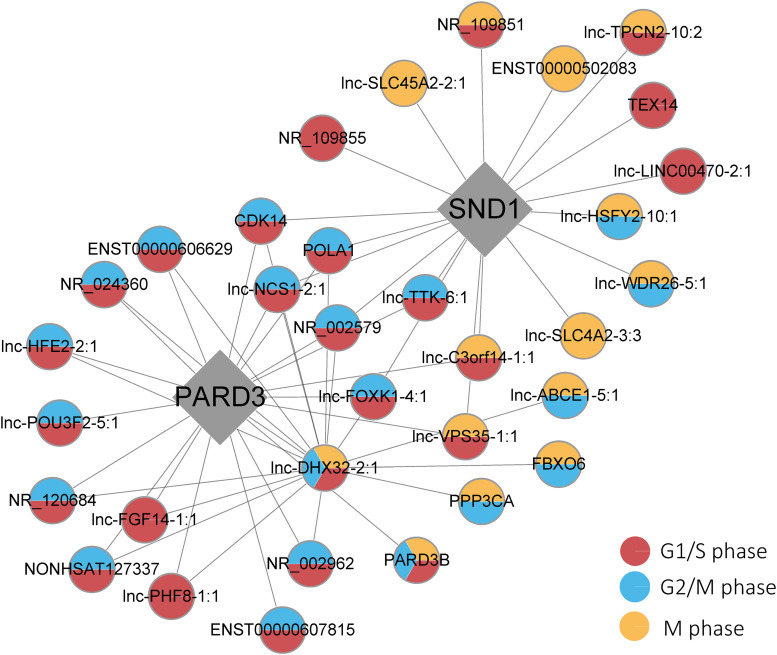
Key driver gene subnetworks with the phases of the cell cycle. Red, blue, and yellow circles indicate G1/S, G2/M, and M phases, respectively. Diamonds represent the key driver genes.

Finally, three top key driver genes, including PARD3 (FDR = 1.46 × 10^–22^), SND1 (FDR = 5.24 × 10^–8^), and lnc-DHX32-2:1 (FDR = 1.43 × 10^–17^), were particularly highlighted in the category of G2/M, G1/S, and M phases and mathematically identified as the causal modulators of the regulatory state of the functionally relevant gene group based on prior knowledge (Figure 5). Moreover, several genes showed great connectivity in our causal gene network and interrelated with every single key driver gene, including CDK14, POLA1, lnc-NCS1-2:1, and lnc-FOXK1-4:1 (all FDR < 0.05 in those phases). Furthermore, lnc-DHX32-2:1 and PARD3B existed in three categories in our list, illustrating their potential relationship within the probabilistic causal gene network, which might suggest their complex cellular context. Additionally, some significantly differentially expressed genes existed in the network, such as FBXO6 and ENST00000607815. It was also revealed that most genes might participated in multiple phases regulation, especially in the progression G1/S and G2/M phases.

### Validation by Real-Time RT-PCR

**FIGURE 6:**
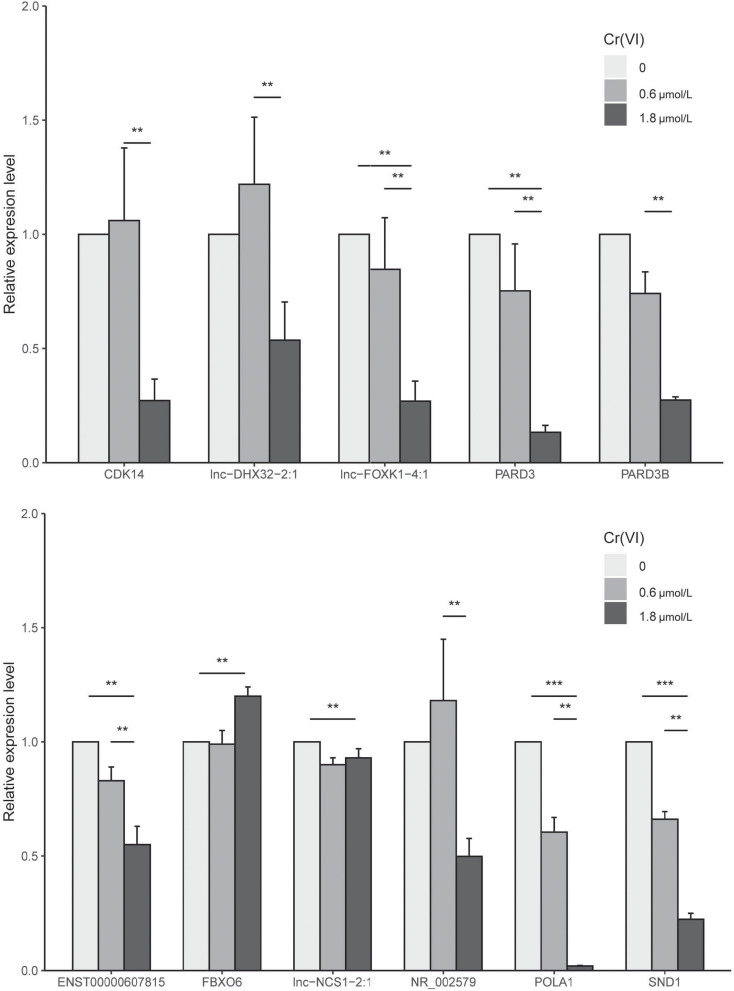
Results of RT-PCR analysis of 11 genes in the network. ** indicates significantly different at 0.05, *** indicates significantly different at 0.001.

To determine whether the expression patterns of these genes could be recapitulated, we selected ENST00000607815, lnc-NCS1-2:1, FBXO6, CDK14, lnc-DHX32-2:1, lnc-FOXK1-4:1, PARD3, PARD3B, POLA1, SND1, NR_002579, TEX14, lnc-C3orf14-1:1 for real-time RT-PCR analysis (Figure 6). The results found 11 different expressed genes in our network, including the key driver genes SND1 and PARD3, which had significantly change between control and 1.8 μmol/L group. Eight genes which both connected with PARD3 and SND1, including lnc-NCS1-2:1, FBXO6, CDK14, lnc-DHX32-2:1, lnc-FOXK1-4:1, POLA1, NR_002579 and ENST00000607815 could be divided into three groups, (1) six of them located in G1/S and G2/M phase sub-nodes, (2) lnc-DHX32-2:1 related with all cell cycle phases change, and (3) FBXO6 was in G2/M or M phase node. Among these genes, lnc-FOXK1-4:1, POLA1 and ENST00000607815 had the most significant change in Cr group, indicating the potential effect on G1/S and G2/M phases.

## Discussion

The present study provided a regulatory network containing protein-coding genes and lncRNAs, including PARD3, SND1, POLA1, FBXO6, lnc-NCS1-2:1, lnc-FOXK1-4:1, lnc-DHX32-2:1, and ENST00000607815, which revealed the key regulatory mechanisms in different cell cycle phases after exposure to Cr(VI). Because our *in vitro* experiment was based on relatively low doses of Cr, it can be referenced as a common environmental exposure level that mimics the damage on human respiratory epithelial cells. Our results regarding the cell cycle were consistent with previous findings that Cr exposure could induce cell cycle changes by regulating checkpoint pathways to control the order and timing of cell cycle phase transition (Alcedo et al., 1994; Chiu et al., 2010; Nickens et al., 2010; Proctor et al., 2014; Rager et al., 2019), even under very low concentrations (Ha et al., 2004; Wakeman et al., 2004). In particular, the bioinformatics approach we used provided us with potential candidate lncRNAs that might play important roles in Cr(VI) exposure via the regulation of different cell cycle phases.

In the past two decades, the number of papers related to the cell cycle has rapidly increased (Kastan and Bartek, 2004; Malumbres and Barbacid, 2009) because of the complex and diverse processes involved in the cellular responses to DNA damage. Both endogenous and exogenous sources that cause DNA damage are considered major contributors in the development progress of human cancers, and thus it is reasonable to speculate that defects in cell cycle genes have critical significance in increasing the respiratory cancer risk for occupational exposure to Cr(VI) (Seidler et al., 2013). Previous transcriptomic-based studies have indicated that genes relevant to cell proliferation and DNA repair showed differential expression patterns after Cr(VI) exposure. Among these pathways, the effects of inhibitors of ATM activation have received the most attention because of the observed toxicological importance in activation of the DNA damage-responsive kinase ATM after Cr(VI) exposure (Luczak et al., 2016).

However, this pathway did not appear in our experimental study, which may be because of the threshold of checkpoint activation or DNA-damage response (Liang et al., 2014). Our results offered novel candidate regulatory genes using mathematical algorithms and cells treated with relatively low doses (< 2 μmol/L) of Cr(VI), which altered the cell cycle phases in a concentration-dependent manner. The high level of complexity in cell cycle regulation after exposure to potassium dichromate provides a great opportunity to discover novel factors in several signaling and response pathways to address the specific nature of cell damage. To minimize the adverse effects of DNA damage situation, DNA repair is a mechanism that allows cells to properly repair these defects. This is exemplified in the results of a study involving PARD3 and PARD3B, which encode proteins belonging to the Par-3 family of cell polarity regulators, that play key roles in asymmetrical cell division and polarized cell growth. Studies showed that Par-3 complexes could regulate DNA-PK directly or via Ku70 which could affect the progress of DNA double-strand break repair (Fang et al., 2007), thus indicated that Par-3 enhanced NHEJ and HR pathway required for efficient DNA repair in G2 and M phases. Besides, the loss or attenuation of epithelial polarity is a hallmark of epithelial to mesenchymal transformation (EMT) (Thiery, 2003), indicating the possibility of regulating cell cycle and enhancing the genetic instability by PARD3 or PARD3B (Supplementary Figures 2, 3). Together, this suggests the possibility that Par-3 is involved in cancer development and progression in the G1/S or G2/M phase. Moreover, the gene SND1 (staphylococcal nuclease and tudor domain containing 1) in the center of the network mediated miRNA decay of both protein-free and AGO2-loaded miRNAs and also regulated mRNAs involved in G1-to-S phase transition (Elbarbary et al., 2017). Extensive research also supports the conclusion that SND1 is an oncoprotein in a variety of cancers involving multiple processes (Jariwala et al., 2017; Xin et al., 2019) and that it also acts as an essential effector in promoting EMT in cancer (Xin et al., 2019; He et al., 2020). In addition, FBXO6 was found to closely interact with Chk1 (Tu et al., 2017), which indirectly affected the ATR-Chk1 signal axis by various kinds of DNA damage insults, including replication stress, inter-strand cross-link (ICL), and DSBs (Jazayeri et al., 2006). Similarly, a study demonstrated that FBXO6 correlated with the survival of non-small cell lung cancer (NSCLC) patients (Cai et al., 2019). CDK14 plays fundamental role in regulating the G2/M phase of the cell cycle by mediating the phosphorylation of LRP5/6 in the Wnt signaling pathway (Wang et al., 2016). Correspondingly, CDK14 played an important role in lung tumorigenesis. For example, the cigarette smoke-induced downregulation of CDK14 in lung cells correlated with β-catenin levels, suggesting impaired Wnt signaling (Pollack et al., 2015). Another study also suggested that CDK14 is regulated by SNHG15 by competitively sponging miR-486, which contributed to NSCLC tumorigenesis (Jin et al., 2018).

As RNAs do not function alone, we conducted WGCNA and probabilistic causal gene analyses to model underlying molecular relationships and causal gene connections, and used mathematical approaches and key driver analysis (KDA) to prioritize key drivers of the genotoxicity of Cr(VI). WGNCA analysis was conducted to narrow down the extent of related lncRNAs with our selected genes with a dose-response relationship, and KDA was used to identify the key regulator within the co-expression network lncRNAs, including ENST00000607815, lnc-NCS1-2:1, lnc-DHX32-2:1, and lnc-FOXK1-4:1, were verified as the important lncRNAs in our network. One of the main regulators of lnc-DHX32-2:1, which targeted for gene ADAM12, has been implicated in a variety of biological processes, including lung cancer and the development of giant cell tumors, and positively involved in the regulation of the MAPK/ERK pathway. Moreover, the MAPK/ERK pathway plays an important role in integrating external signals to promote the transition of G1 to S phase, while ERK activation downstream of mitogen-induced Ras signaling is sufficient to alleviate cell cycle arrest and allow cells to progress to the S-phase (Chambard et al., 2007). This is consistent with a previous study in which Cr(VI) activated ERK signaling pathways (Chuang et al., 2000), although no cytotoxicity effects were observed. Indeed, our analysis identified lncRNAs as interesting candidates involved in cell-cycle gene regulation, but all guilt-by-association approaches must be treated with caution and recognized as hypothesis-generating research. Additionally, all exploratory data analysis techniques require extensive targeted studies to confirm suggested molecular networks and the potential mechanisms.

Finally, our study revealed that cells exhibited cytotoxicity even in the low-dose exposure group. The choice of relatively low concentrations based on the conclusion of previous studies showed that BEAS-2B cells exhibited increased proliferation in the presence of 1 μM (Nickens et al., 2010; Cerveira et al., 2014), whereas other studies showed slightly decreased proliferation following treatment with low micromolar range concentrations after stimulating human bronchial epithelial cells (Abreu et al., 2018; Hu et al., 2019). The 2 μM concentration was considered to cause significant cytotoxic effects and alterations of the cell cycle. Therefore, increasing the sample size and number of doses would allow for more convincing conclusions. Our research design follows the principle of Toxicity Testing in the 21st Century, which emphasized the usage of cell lines, computational modeling, and bioinformatics approaches to explain cell response progress (Krewski et al., 2010; Seidler et al., 2013). This research also implied that the DNA repair or immune defense mechanisms might involve greatly to cope with the toxicity in relatively low level, which is different from the proved results, and can help assess the dose-response characteristics in exploring the perturbation of the adverse pathways (Krewski et al., 2010).

The current study had several limitations. First, the use of single omics technology reveals the cellular transcription responses patter on Cr, which might have limitation in explaining the results in the aspect of functional outcomes. Subsequently, the transcriptome experiment conducted by microarrays with high background levels owing to cross-hybridization confines the accuracy of expression estimation, particularly for the transcripts in low abundance (Zecevic et al., 2009). Finally, this study based on a single cell line that cannot depict the whole picture of complicated toxic responses in human pulmonary bronchial. Thus, further research on different human cell as well as the *in vivo* approaches, such as cell cycle checkpoint and related protein analysis, that aiming to test the regulation of cyclin-dependent kinases secretion with knock-out amplification these genes can offer evidence on how they activate or inhibit cell cycle phases, which can be used to explain the carcinogenicity and Cr(VI) toxicity. Another potential limitation is that repeated experiments under different passages and freeze downs are needed for further analysis. In the future, we will apply this method to other cell lines, for example, HBECs and hTERT-immortalized lung Cells. With the development on 3D-cell-culture model *in vitro* (Gunness et al., 2013; Amann et al., 2014). Our further research will try to analysis the transcriptome character in different condition with the 3D Cultures method to construct lung model (Rayner et al., 2019). Nonetheless, further integrated proteomics, epigenomics or the RNA modifications analysis on multi-omics level and time series studies with RNA-seq methods are needed to confirm the mechanisms of toxicity involved in the cell cycle alterations induced by Cr(VI).

## Conclusion

We designed a novel computational workflow that showed the toxicity effects of Cr(VI) on the cell cycle. We identified gene networks and candidate lncRNAs and mRNAs in relatively low dose range suggesting that the gene sets could provide a clue for toxic responses in cell cycle regulation to environmental and occupational Cr(VI) exposures.

## Data Availability Statement

The datasets presented in this study can be found in online repositories. The names of the repository/repositories and accession number(s) can be found below: https://github.com/reefur/Chromium-transcriptome/blob/main/database_cr.xlsx.

## Author Contributions

PZ and ZW participated in the study design, statistical and bioinformatics analysis, and manuscript preparation. SH, HF, and FH conducted the experiment. DZ and CL provided assistance with gene set network analysis. GH, ZC, ZW, TW, and GJ reviewed the figures and manuscript. All authors contributed to the article and approved the submitted version.

## Conflict of Interest

The authors declare that the research was conducted in the absence of any commercial or financial relationships that could be construed as a potential conflict of interest.
