# Supplementary material for: A Novel Transcriptome Integrated Network Approach Identifies the Key Driver lncRNA Involved in Cell Cycle With Chromium (VI)-Treated BEAS-2B Cells
Source: Front Genet. 2021 Jan 13;11:597803. doi: 10.3389/fgene.2020.597803 (PMC7838612; doi:10.3389/fgene.2020.597803)
Supplement: Supplementary file 1 [file Data_Sheet_1.zip › Supplementary Files/Figure 1.pdf]

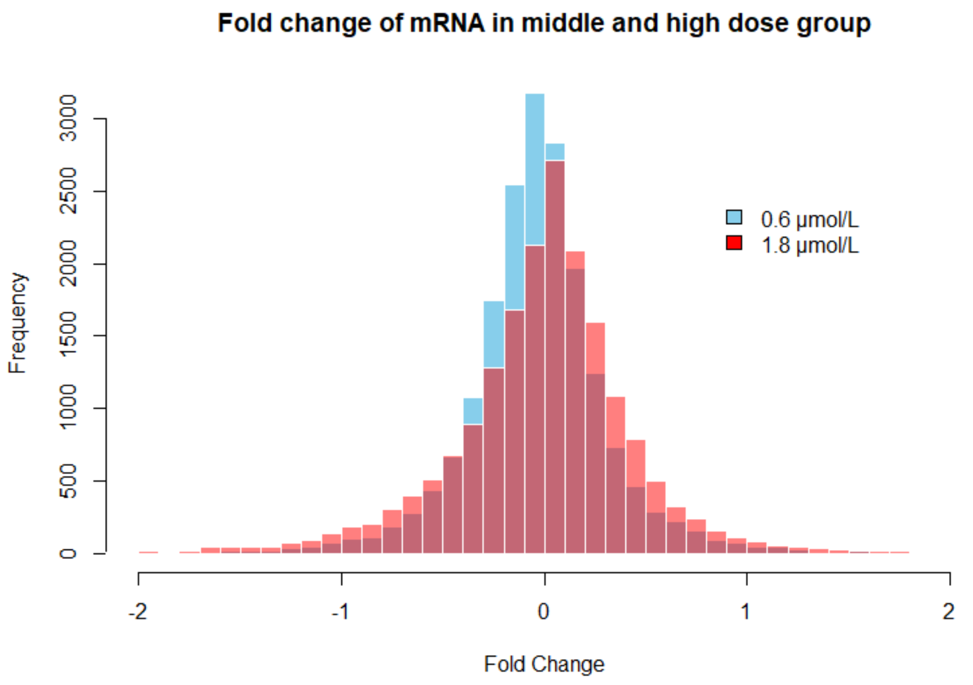

A

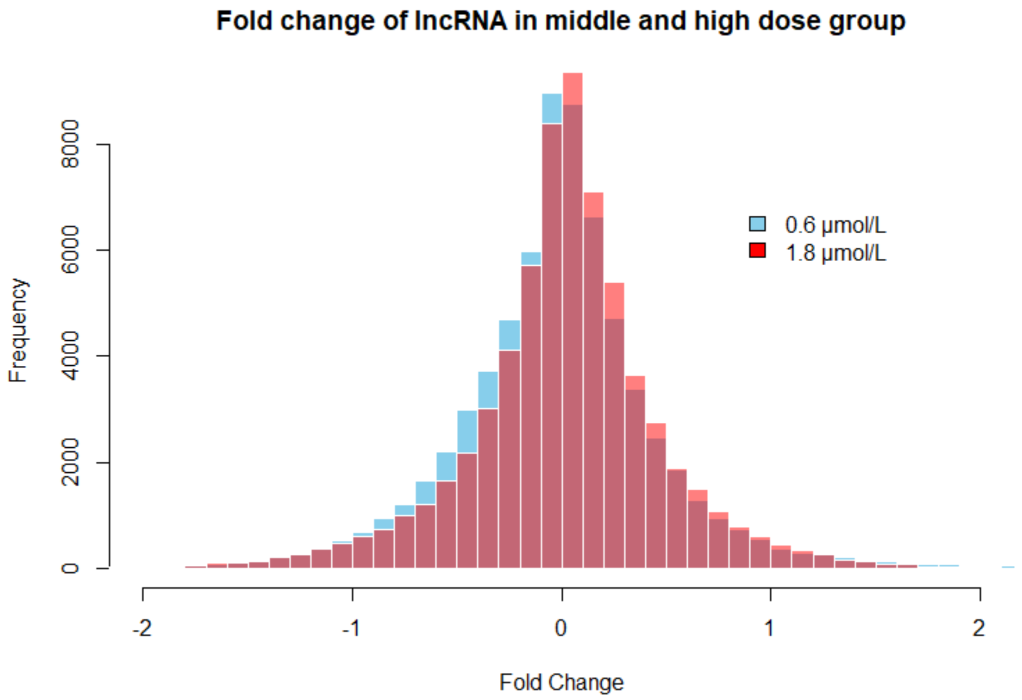

b

Supplementary Figure 1 The frequency of mRNA and lncRNA expression levels between the two groups.
