# Supplementary material for: A Novel Transcriptome Integrated Network Approach Identifies the Key Driver lncRNA Involved in Cell Cycle With Chromium (VI)-Treated BEAS-2B Cells
Source: Front Genet. 2021 Jan 13;11:597803. doi: 10.3389/fgene.2020.597803 (PMC7838612; doi:10.3389/fgene.2020.597803)
Supplement: Supplementary file 1 [file Data_Sheet_1.zip › Supplementary Files/Table 1.docx]

Supplementary Table 1 Differentially expressed genes in all dose groups

| Dose group | Regulation | mRNA  (N=18833) | lncRNA  (N=68104) | Sum |
| --- | --- | --- | --- | --- |
| low dose group |  |  |  |  |
|  | upregulation | 6 | 0 | 6 |
|  | downregulation | 11 | 0 | 11 |
| middle dose group |  |  |  |  |
|  | upregulation | 39 | 39 | 78 |
|  | downregulation | 6 | 45 | 51 |
| high dose group |  |  |  |  |
|  | upregulation | 229 | 417 | 646 |
|  | downregulation | 484 | 287 | 771 |
