# Supplementary figures and images for: A Novel Transcriptome Integrated Network Approach Identifies the Key Driver lncRNA Involved in Cell Cycle With Chromium (VI)-Treated BEAS-2B Cells
Source: Front Genet. 2021 Jan 13;11:597803. doi: 10.3389/fgene.2020.597803 (PMC7838612; doi:10.3389/fgene.2020.597803)

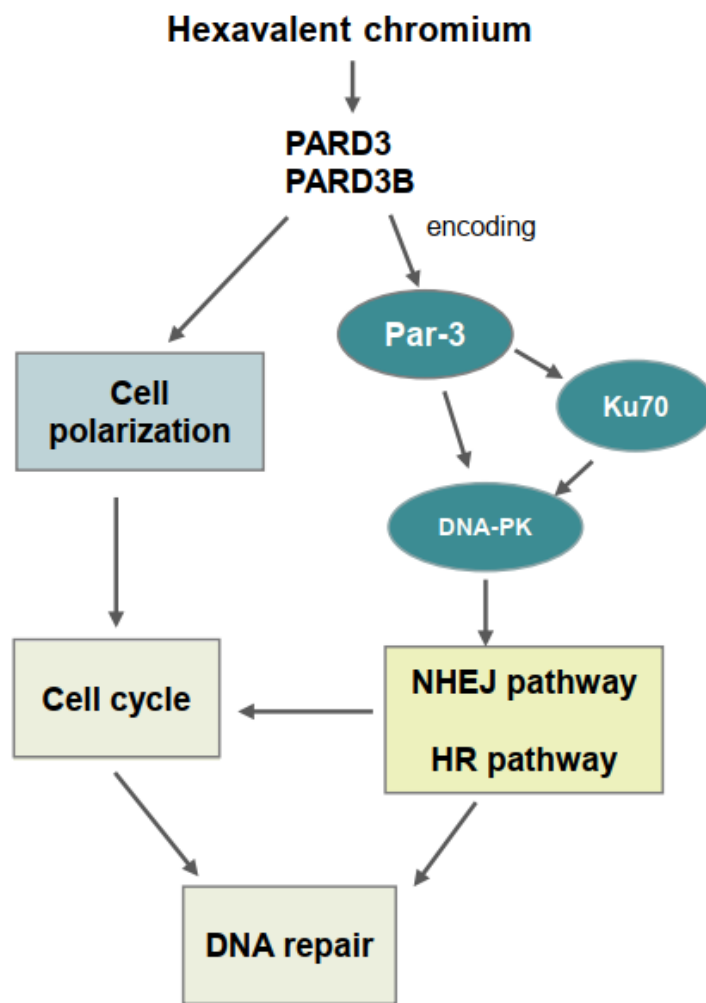

Supplement Figure 3 Pathway map of PARD3 gene in cell cycle and DNA repair

Supplement: Supplementary file 1 [file Data_Sheet_1.zip › Supplementary Files/Figure 3.pdf]
